# Supplementary material for: An ON-type direction-selective ganglion cell in primate retina
Source: Nature. 2023 Oct 25;623(7986):381–6. doi: 10.1038/s41586-023-06659-4 (PMC10632142; doi:10.1038/s41586-023-06659-4)
Supplement: Supplementary file 1 — Reporting Summary [file 41586_2023_6659_MOESM1_ESM.pdf]

nature portfolio

Corresponding author(s): Teresa Puthussery

Last updated by author(s): Aug 25, 2023

Reporting Summary

Nature Portfolio wishes to improve the reproducibility of the work that we publish. This form provides structure for consistency and transparency in reporting. For further information on Nature Portfolio policies, see our Editorial Policies and the Editorial Policy Checklist.

Statistics

For all statistical analyses, confirm that the following items are present in the figure legend, table legend, main text, or Methods section.

n/a	Confirmed	
☐	☒	The exact sample size (*n*) for each experimental group/condition, given as a discrete number and unit of measurement
☐	☒	A statement on whether measurements were taken from distinct samples or whether the same sample was measured repeatedly
☐	☒	The statistical test(s) used AND whether they are one- or two-sided<br>*Only common tests should be described solely by name; describe more complex techniques in the Methods section.*
☒	☐	A description of all covariates tested
☐	☒	A description of any assumptions or corrections, such as tests of normality and adjustment for multiple comparisons
☐	☒	A full description of the statistical parameters including central tendency (e.g. means) or other basic estimates (e.g. regression coefficient) AND variation (e.g. standard deviation) or associated estimates of uncertainty (e.g. confidence intervals)
☐	☒	For null hypothesis testing, the test statistic (e.g. *F*, *t*, *r*) with confidence intervals, effect sizes, degrees of freedom and *P* value noted<br>*Give P values as exact values whenever suitable.*
☒	☐	For Bayesian analysis, information on the choice of priors and Markov chain Monte Carlo settings
☒	☐	For hierarchical and complex designs, identification of the appropriate level for tests and full reporting of outcomes
☒	☐	Estimates of effect sizes (e.g. Cohen's *d*, Pearson's *r*), indicating how they were calculated

Our web collection on statistics for biologists contains articles on many of the points above.

Software and code

Policy information about availability of computer code

Data collection	Zen 2 software (Zeiss), SciScan (v1.3, Scientifica), Zeiss Axiovision software (v 4.8.0.0), ScanImage Basic v2021.01.0 (MBF Biosciences), PsychoPy 2022.2.0 or earlier
Data analysis	ImageJ (1.53q, NIH, USA), Igor Pro (version 9.0.0.10, Wavemetrics), Imaris 9.8.2 (Oxford Instruments), sjedrp v0.12, Simple Neurite Tracer Plugin for ImageJ (4.1.9), bUnwarpJ 2.6.12 plugin for ImageJ, custom ImageJ macros, custom Python code (Google Colaboratory)

For manuscripts utilizing custom algorithms or software that are central to the research but not yet described in published literature, software must be made available to editors and reviewers. We strongly encourage code deposition in a community repository (e.g. GitHub). See the Nature Portfolio guidelines for submitting code & software for further information.

Data

Policy information about availability of data

All manuscripts must include a data availability statement. This statement should provide the following information, where applicable:

- Accession codes, unique identifiers, or web links for publicly available datasets
- A description of any restrictions on data availability
- For clinical datasets or third party data, please ensure that the statement adheres to our policy

GEO datasets: GEO:GSE118480 (macaque), GEO:GSE148077 (human), GEO:GSE137400 (mouse)
All raw data generated in this study has been deposited in a publicly available data repository.

Human research participants

Policy information about studies involving human research participants and Sex and Gender in Research.

Reporting on sex and gender	N/A
Population characteristics	N/A
Recruitment	N/A
Ethics oversight	N/A

Note that full information on the approval of the study protocol must also be provided in the manuscript.

Field-specific reporting

Please select the one below that is the best fit for your research. If you are not sure, read the appropriate sections before making your selection.

☒ Life sciences ☐ Behavioural & social sciences ☐ Ecological, evolutionary & environmental sciences

For a reference copy of the document with all sections, see nature.com/documents/nr-reporting-summary-flat.pdf

Life sciences study design

All studies must disclose on these points even when the disclosure is negative.

Sample size	No statistical methods were used to determine sample size a priori. Sample size was determined based on similar experiments on sparse primate retinal ganglion cell types. We used appropriate statistical tests to determine statistical significance given the sample size.
Data exclusions	For calcium imaging, cells that were deemed unresponsive based on a response threshold (dF/F < 1.5 s.d. above baseline) were excluded from further analysis.
Replication	All experiments were repeated on multiple cells from different animals as indicated in the manuscript.
Randomization	No experimental groups were assigned in this study.
Blinding	Data acquisition and analyses were not performed with blinding to the experimental conditions as most experiments did not involve a treatment or perturbation and analyses were automated. In the case of experiments using gabazine, the control and drug trials were analyzed automatically, without consideration of trial conditions.

Reporting for specific materials, systems and methods

We require information from authors about some types of materials, experimental systems and methods used in many studies. Here, indicate whether each material, system or method listed is relevant to your study. If you are not sure if a list item applies to your research, read the appropriate section before selecting a response.

Materials & experimental systems

n/a	Involved in the study	
☐	☒	Antibodies
☒	☐	Eukaryotic cell lines
☒	☐	Palaeontology and archaeology
☐	☒	Animals and other organisms
☒	☐	Clinical data
☒	☐	Dual use research of concern

Methods

n/a	Involved in the study	
☒	☐	ChIP-seq
☒	☐	Flow cytometry
☒	☐	MRI-based neuroimaging

Antibodies

Antibodies used	Full details of antibodies used in this study have been provided with the submission in Extended Data Table 1
Validation	Full details of antibody validation has been provided in Extended Data Table 1. Data supporting validation of BNC2 antibodies are

Validation	provided in Extended Data Fig. 7. We tested multiple antibodies to compare localization patterns and confirmed that expression matched that expected based on orthogonal methods (single-cell RNA sequencing).

Animals and other research organisms

Policy information about studies involving animals; ARRIVE guidelines recommended for reporting animal research, and Sex and Gender in Research

Laboratory animals	Mus muscularis: B6J.Cg-Gt(ROSA)26Sortm95.1(CAG-GCaMP6f)Hze/MwarJ, (JAX strain #:028865; RRID:IMSR_JAX:028865), B6J.129S6(FVB)-Slc17a6tm2(cre)Lowl/MwarJ, (JAX strain #028863; RRID:IMSR_JAX:028863), C57BL/6J (JAX strain#:000664; RRID:IMSR_JAX:000664). Age: 6 - 26 weeks. Animals had ad libitum access to food and water and were kept on a 12/12 h light/dark cycle. Ambient temperature and humidity were maintained at 20-22°C and 50-60%.<br>Rhesus Macaque: Macaca mulatta. Age 1.36-19.17 years.
Wild animals	No wild animals were used in this study.
Reporting on sex	Macaque eyes were obtained from 6 males and 5 females. Mouse eyes were from 1 male and 2 females. Data were not disaggregated for sex given the small number of animals used in each experiment.
Field-collected samples	There were no field-collected samples in this study.
Ethics oversight	All mouse procedures were approved by the Animal Care and Use Committee of the University of California, Berkeley. Macaque tissue collection procedures at UC Berkeley were approved by the UC Berkeley Animal Care and Use Committee and conducted in accordance with National Research Council guidelines. Tissues from Oregon & California National Primate Centers were collected immediately post-mortem from animals used for unrelated studies.

Note that full information on the approval of the study protocol must also be provided in the manuscript.
